# Electrocatalytic Behavior of an Amide Functionalized Mn(II) Coordination Polymer on ORR, OER and HER

**DOI:** 10.3390/molecules27217323

**Published:** 2022-10-28

**Authors:** Anup Paul, Kristina Radinović, Susanta Hazra, Dušan Mladenović, Biljana Šljukić, Rais Ahmad Khan, Maria Fátima C. Guedes da Silva, Armando J. L. Pombeiro

**Affiliations:** 1 Centro de Química Estrutura, Institute of Molecular Sciences, Instituto Superior Técnico, Universidade de Lisboa, Av. Rovisco Pais, 1049-001 Lisboa, Portugal; 2 University of Belgrade, Faculty of Physical Chemistry, Studentski Trg 12-16, 11158 Belgrade, Serbia; 3 Center of Physics and Engineering of Advanced Materials, Instituto Superior Técnico, Universidade de Lisboa, 1049-001 Lisbon, Portugal; 4 Department of Chemistry, College of Science, King Saud University, P.O. Box 2455, Riyadh 11451, Saudi Arabia; 5 Departamento de Engenharia Química, Instituto Superior Técnico, Universidade de Lisboa, Av. Rovisco Pais, 1049-001 Lisboa, Portugal; 6 Research Institute of Chemistry, Peoples’ Friendship University of Russia (RUDN University), 6 Miklukho-Maklaya Street, 117198 Moscow, Russia

**Keywords:** oxygen reduction reaction, oxygen evolution reaction, hydrogen evolution reaction, manganese, coordination polymer

## Abstract

The new 3D coordination polymer (CP) [Mn(L)(HCOO)]_n_ (**Mn-CP**) [L = 4-(pyridin-4-ylcarbamoyl)benzoate] was synthesised via a hydrothermal reaction using the pyridyl amide functionalized benzoic acid HL. It was characterized by elemental, FT-IR spectroscopy, single-crystal and powder X-ray diffraction (PXRD) analyses. Its structural features were disclosed by single-crystal X-ray diffraction analysis, which revealed a 3D structure with the monoclinic space group P21/c. Its performance as an electrocatalyst for oxygen reduction (ORR), oxygen evolution (OER), and hydrogen evolution (HER) reactions was tested in both acidic (0.5 M H_2_SO_4_) and alkaline (0.1 M KOH) media. A distinct reduction peak was observed at 0.53 V vs. RHE in 0.1 M KOH, which corresponds to the oxygen reduction, thus clearly demonstrating the material’s activity for the ORR. Tafel analysis revealed a Tafel slope of 101 mV dec^−1^ with mixed kinetics of 2e^−^ and 4e^−^ pathways indicated by the Koutecky–Levich analysis. Conversely, the ORR peak was not present in 0.5 M H_2_SO_4_ indicating no activity of **Mn-CP** for this reaction in acidic media. In addition, **Mn-CP** demonstrated a noteworthy activity toward OER and HER in acidic media, in contrast to what was observed in 0.1 M KOH.

## 1. Introduction

In recent years, the energy crisis and environmental pollution have driven the development of new efficient and “green” technologies for renewable energy conversion and storage, such as water splitting, metal–air batteries and fuel cells [1,2,3]. These technologies are primarily centered around three key electrocatalytic reactions: hydrogen evolution reaction (HER), oxygen evolution reaction (OER), and oxygen reduction reaction (ORR), whose efficiency largely depends on the performance of catalysts [4,5]. Currently, efficient catalysts for the ORR and HER are considered to be Pt and its derivatives, while Ir/Ru oxides are considered to be the best catalysts for OER. However, the scarcity and high price of precious metals are critical barriers for large-scale practical applications [6,7]. Researchers around the world have addressed their studies on HER, OER and ORR catalysts, such as transition metal carbides (TMC) [8,9,10], carbon-based hybrids [11,12,13], and graphene doped with heteroatoms [14,15,16]. However, the main challenge is to find a non-precious metal-based catalyst with a high catalytic performance comparable to the already mentioned Pt and Ir/Ru oxides.

Metal–organic frameworks (MOFs), also known as coordination polymers (CPs), are materials with broad structural diversity that over the years have developed into an intriguing class of hybrid materials that have been incorporated into many applications [17,18,19,20,21]. In addition, they offer a great possibility of developing multifunctional electrocatalysts [22,23,24]. For example, Zhao et al. reported the catalytic activity of two-dimensional M_3_(HITP)_2_ (where HITP = 2,3,6,7,10,11-hexaiminotriphenylene) with M = Fe, Co, Ni, Cu and Zn for HER/OER and OER/ORR as bifunctional electrocatalysts [25]. The multifunctional catalytic activity of this catalyst is attributed to the synergistic effect of M and organic ligands and the electrocatalytic performance of M_3_(HITP)_2_ monolayers that can be modulated by changing the M atoms. Their calculations showed that a monolayer of Cu_3_(HITP)_2_ is a promising HER/OER bifunctional catalyst for water splitting with low overpotentials of only −0.02/+0.75 V, which can be compared to precious metal catalysts. On the other hand, the Cu_3_(HITP)_2_ monolayer represents an OER/ORR bifunctional catalyst with overpotentials of 0.36/0.73 V [25].

Sun and coworkers reported that MBene [where MBenes = 2D metal borides (Mo_2_B_2_)]-supported single-atom catalysts (SACs) by embedding a series of transition metal atoms in the Mo vacancy (M@Mo_2_B_2_; M = Ti, V, Cr, Mn, Fe, Co, Ni and Cu) as electrocatalysts in HER, OER and ORR [26]. Among these, Ni@Mo_2_B_2_ and Cu@Mo_2_B_2_ have shown good structural stability and excellent metal conductivity, and were tested as bifunctional electrocatalysts for HER/OER and OER/ORR. Ni@Mo_2_B_2_ as HER/OER bifunctional electrocatalyst showed a lower overpotential (0.52 V) than IrO_2_ (0.56 V) for OER. In addition, Cu@Mo_2_B_2_ as OER/ORR bifunctional electrocatalyst displayed a lower overpotential (0.34 V) than Pt (0.45 V) for ORR and a lower overpotential (0.31 V) than IrO_2_ for OER [26].

Very recently, we have reported a couple of coordination polymers derived from an amide functionalized ligand, which were found to be effective for water splitting reactions [19]. The presence of the amide backbone in combination with the metal center provided a promising material for supercapacitors and electrocatalysts for water splitting [19]. We noticed that the amide-functionalized ligands in MOFs/CPs exhibit a good stability, which is one of the most interesting prerequisites for electrocatalyst and supercapacitor properties. Recent studies have also demonstrated that MOFs/CPs equipped with amide-functionalized ligands display a good stability and exhibit relevant electrocatalyst and supercapacitor properties [19,27,28]. Thus, in line with our continued search for the development of new amide functionalized MOFs/CPs as electrocatalysts for energy storage/conversion reactions, herein we probe a novel Mn(II) CP as a trifunctional electrocatalyst for ORR, OER and HER.

## 2. Experimental Section

### 2.1. Materials and Methods

4-Aminopyridine, terepthalic acid, thionylchloride (SOCl_2_), the metal salt Mn(NO)_3_·4H_2_O as well as common organic solvents, were purchased from the Sigma-Aldrich Chemical Co. and used as received. FT-IR spectra were recorded on a Bruker Vertex 70 instrument in KBr pellets. ^1^H (300 MHz) and ^13^C (75.45 MHz) NMR spectra were obtained at room temperature (RT) on a Bruker Avance II + 300 (UltraShieldTMMagnet) spectrometer using tetramethylsilane [Si(CH_3_)_4_] as an internal reference. Carbon, hydrogen and nitrogen elemental analyses were carried out by the Microanalytical Service of the Instituto Superior Técnico. Single crystal X-ray diffraction data were collected using a Bruker APEX-II PHOTON 100 diffractometer with graphite monochromated Mo-Kα (λ = 0.71069) radiation. Powder X-ray diffraction (PXRD) was conducted in a D8 Advance Bruker AXS (Bragg-Brentano geometry) theta-2-theta diffractometer, with copper radiation (Cu-Kα, λ = 1.5406 Å) and a secondary monochromator, operated at 40 kV and 40 mA. A flat plate configuration was used, and the typical data collection range was between 5 and 40. Thermal properties were studied using a Perkin-Elmer Instrument system (STA6000) at a heating rate of 10 °C/min under a dinitrogen atmosphere and a flow rate of 30 mL/min. Sorption parameters were obtained from nitrogen adsorption isotherms at 77 K, using a Micromeritics ASAP 2060 SurfaceArea Analyzer with prior degassing at 130 °C for 12 h.

### 2.2. Synthesis and Characterization

#### 2.2.1. Synthesis of 4-(Pyridin-4-ylcarbamoyl) Benzoic Acid (HL)

Synthesis of the pro-ligand **HL** was carried out in accordance with the previously reported procedure [29].

#### 2.2.2. Synthesis of [Mn(L)(HCOO)]_n_ (**Mn-CP**)

In a 5 mL glass vial, the pro-ligand **HL** (0.10 g, 0.41 mmol) was added to 2 mL of DMF and heated until the solution became homogeneous. With the addition of Mn(NO_3_)_2_·4H_2_O (0.10 g. 0.41 mmol), a few drops of 1 M HCl solution were added, and the reaction mixture was placed in a reactor for 48 h at 80 °C. We then separated the white crystals of **Mn-CP** formed at the bottom of the glass vial by filtration, washed them with deionized water and DMF, and then dried them in air. The white crystals of **Mn-CP** are insoluble in common solvents like CH_2_Cl_2_, CHCl_3_, MeOH, EtOH, ACN, DMF, DMSO. Isolated yield = 0.058 g (58%). Anal. Calcd for C_14_H_10_MnN_2_O_5_: C, 49.29; H, 2.95; N, 8.21; Found C, 49.36; H, 3.08; N, 8.37. FT-IR (cm^−1^): 1562 cm^−1^ *ν_sy_*(OCO), 1345 cm^−1^ *ν_asy_*(OCO).

#### 2.2.3. Crystal Structure Determination

X-ray quality crystals of **Mn-CP** were immersed in cryo-oil, and a selected one was mounted in a Nylon loop and measured at 150 K. Intensity data were collected using a Bruker APEX II SMART CCD diffractometer with graphite monochromated Mo-Kα (λ = 0.71073) radiation. Cell parameters were obtained with Bruker SMART [30] software and refined with Bruker SAINT [30] on all the observed reflections. Absorption corrections were made by the multi-scan method (SADABS) [30]. Structures were solved by direct methods using the SHELXS-2014 package and refined with SHELXL-2014/7 [31]. Calculations were performed using the WinGX System-Version 2014.1 [32]. The hydrogen atoms attached to carbon and N-amide atoms were inserted at geometrically calculated positions and included in the refinement using the riding-model approximation. The least square refinements with anisotropic thermal motion parameters for all the non-hydrogen atoms were employed. Crystallographic data are summarized in Tables S1 and S2. CCDC 2,206,351 contains the supplementary crystallographic data for this paper. These data can be obtained free of charge from The Cambridge Crystallographic Data Centre via www.ccdc.cam.ac.uk/data_request/cif.

#### 2.2.4. Electrochemical Measurements

The catalytic ink was made by dispersing 5 mg of the catalyst into 825 µL of a solution containing 450 µL of distilled water, 300 µL of absolute ethanol, and 75 μL of Nafion (0.5%). A small amount of Vulcan (0.4 mg Cabot Vulcan XC-72R) was added in order to increase the low conductivity of **Mn-CP**. The ink was ultrasonically mixed for 30 min and then 20 µL of ink was loaded onto a polished glassy carbon rotating disc electrode (RDE, 0.19625 cm^2^). The electrode was dried by blowing high purity N_2_ over it. For comparison purposes, catalytic ink was prepared with pure **Mn-CP** as well as with pure Vulcan.

All electrochemical measurements were performed at room temperature (25 °C) using Gamry Interface 1010 galvanostat/potentiostat with a standard three-electrode glass cell connected to a Gamry rotator (Gamry RDE710 Rotating Electrode). Counter and reference electrodes were employed with a graphite rod and saturated calomel electrode (SCE), respectively. All potentials have been converted (and presented) to the reversible hydrogen electrode (RHE) scale using the equation E(RHE) = E(SCE) + 0.242 + 0.059 pH.

The atmosphere was controlled by bubbling high-purity gases (O_2_ or N_2_, Messer, 99.9995 vol.%) into 0.1 M KOH (Sigma-Aldrich, Taufkirchen, Germany) or 0.5 M H_2_SO_4_ (Sigma-Aldrich, Taufkirchen, Germany) electrolyte solution. Electrochemical characterization and investigation of catalytic activity of the sample for ORR, HER and OER were performed using standard electrochemical methods: cyclic voltammetry (CV), linear sweep voltammetry (LSV), electrochemical impedance spectroscopy (EIS), and chronoamperometry. Voltammograms were recorded at a scan rate of 20 mV s^−1^ at rotation speeds of 0, 300, 600, 900, 1200, 1800, 2400, and 3600 rpm. Voltammograms were also recorded in an N_2_-saturated solution at different scan rates from 10 mV s^−1^ to 100 mV s^−1^. The stability and the selectivity of the catalyst were tested in O_2_-saturated 0.1 M KOH in chronoamperometric mode at a constant potential of 0.56 V. OER and HER polarization curves were recorded in 0.1 M KOH or 0.5 M H_2_SO_4_ at a scan rate of 20 mV s^−1^ at 1200 rpm. Electrochemical impedance spectroscopy (EIS) was carried out in the frequency range of 100 kHz to 0.1 Hz, with 10 mV amplitude under both OER and HER polarization conditions in alkaline and acidic media. Potentials used were −0.4 V for HER and 1.7 V for OER conditions in alkaline medium, and −0.6 V for HER and 1.6 V for OER conditions in acidic media. A stability test under OER (constant potential of 1.4 V) and HER (constant potential of −0.5 V) conditions was done in 0.5 M H_2_SO_4_ in chronoamperometric mode during 10 h.

## 3. Results and Discussion

### 3.1. Synthesis and Characterisation

**Scheme 1:**
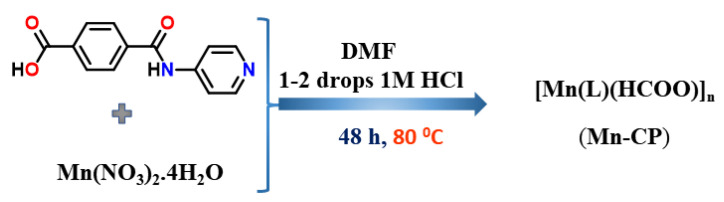
Synthesis of Mn-CP.

The pro-ligand **HL** was synthesized using the procedure previously described [29]. As shown in Scheme 1, [Mn(L)(HCOO)]_n_ (**Mn-CP**) was produced hydrothermally by reacting the pro-ligand **HL** with Mn(NO_3_)_2_·3H_2_O in DMF and adding a few drops of 1 M HCl acid. A combination of elemental analysis, IR and multinuclear NMR (^1^H and ^13^C) techniques was used to characterize **HL**, all of which agreed with the previous report [29]. On the other hand, **Mn-CP** has been characterized by elemental analysis, IR spectroscopy and single-crystal X-ray diffraction analysis (presented below). In the FT-IR spectrum of the pro-ligand **HL**, a band associated with [*ν*(OCO)] vibration at 1695 cm^−1^ is observed, while in **Mn-CP** strong bands due to asymmetric and symmetric stretching vibrations are observed at 1562 cm^−1^ and 1345 cm^−1^, respectively for the coordinated carboxylate groups [29]. The powder XRD analysis was also performed to verify that **Mn-CP** shares the same crystal features as those of the bulk material (Figure S1 Supplementary Material).

### 3.2. Crystal Structure Analysis

**Figure 1:**
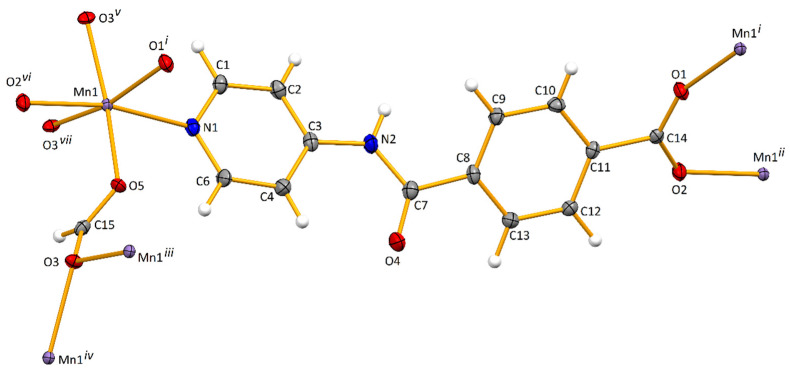
Ellipsoid plot (drawn at 50% probability level) of **Mn-CP** with atom labelling scheme. Symmetry operations to generate equivalent atoms: (*i*) −x,1 − y,−z; (*ii*) −1 + x,y,z; (*iii*) x,1/2 − y,−1/2 + z; (*iv*) 1 − x,−1/2 + y,1/2 − z; (*v*) 1 − x,1/2 + y,1/2 − z; (*vi*) 1 + x,y,z; (*vii*) x,1/2 − y,1/2 + z.

**Figure 2:**
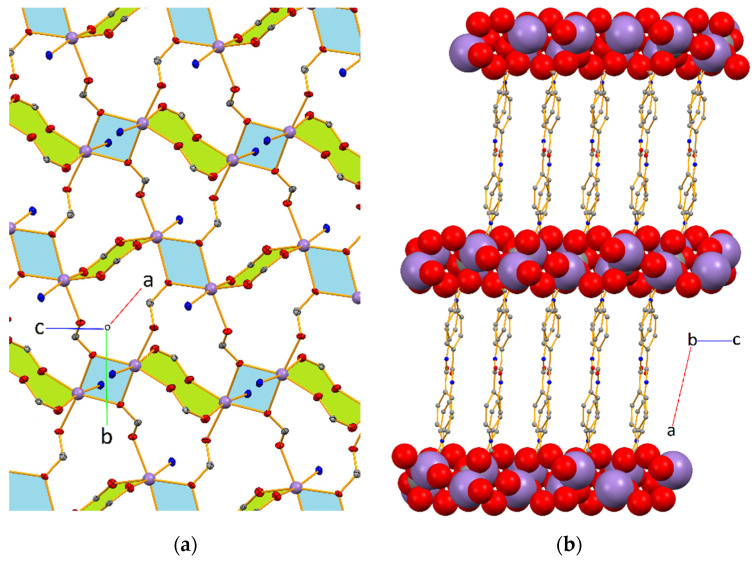
(**a**) A fragment of the infinite 2D layer in Mn-CP revealing chains of corner-shared eight-membered Mn_2_O_4_C_2_ (in green) and four-membered Mn_2_O_2_ (in blue) metallacycles, intercalated with sets of twelve-membered Mn_3_O_6_C_3_ metallacycles (not highlighted). For clarity, the L^–^ ligands are omitted, except for their O- and N-atoms that define the Mn coordination environments; the H-atoms of formate are also excluded. (**b**) A 3D packing is viewed down the crystallographic *b* axis; the Mn cations, the formate and the O_carboxylate_ atoms of the L^–^ ligands are drawn in spacefill model.

The asymmetric unit of [Mn(L)(HCOO)]_n_ (**Mn-CP**; Figure 1) contains a pyridyl-based amide ligand (L^–^) and a formate (HCOO^–^) coordinated to a manganese(II) ion via the N_pyridyl_ (N1) and one of the O_formate_ atoms (O5), respectively. Symmetry expansion reveals that L^–^ acts as a bridging 1κ*N*:2κ*O*:3κ*O*’ chelator and formate as an asymmetric bridging *anti,syn,syn*-1κ*O*:2,3κ*O*’chelator (Figure 1). Therefore, each Mn(II) cation presents a slightly distorted octahedral geometry defined by two O_carboxylate_ and one N_pyridyl_ atoms from three independent L^–^ ligands, as well as three O-atoms from three independent formate groups. In this way, chains of corner-shared eight-membered Mn_2_O_4_C_2_ and four-membered Mn_2_O_2_ metallacycles are formed, interposed by sets of twelve-membered Mn_3_O_6_C_3_ (Figure 2a). The thus formed 2D layers are spread on the *bc* crystallographic plane, are separated by L^–^ pillars and lead to the growth of the structure along the crystallographic *a* axis. The Mn⋯Mn distances in the tetra and hexa metallacycles are of 3.5961(4) and 4.7603(4) Å, respectively. The separation between metal cations bridged by a particular N-ligand assume values of 14.2651(8) and 15.7858(8) Å, as a result of the bending of L^–^ at the level of the C_amide_ and the twisting of the carboxylate group (see below).

The Mn-O and Mn-N bond distances (Table S2) are similar, in the range of 2.1495(10)–2.2663(12) Å, and are comparable to those observed in a reported Mn^II^ derivative with an analogous N-containing moiety [33]. The L^–^ in **Mn-CP** is significantly twisted, as shown by the angle of 37.32° between the least-square planes of the pyridyl and phenyl rings (Table S2). This value is higher than those found in copper(II) and zinc(II) MOFs with the same ligand [29], *viz.* 16.96 and 21.60° in the former, and 10.12° in the latter. The least-square plane of the carboxylate group deviates 22.65° from the one of the phenyl moieties, thus contributing to the significant non-planarity of L^–^ and evading the possibility of efficient π⋯π stacking.

### 3.3. Thermogravimetric Analysis of Mn-CP

To investigate the **Mn-CP** thermal stability, thermogravimetric analysis (TGA) was carried out under dinitrogen between 30 and 800 °C with a heating rate of 10 °C per minute. The formate ligand was lost with a weight loss of 26.21% (calculated = 26.75%) between 183 and 240 °C. The system is thermostable in the 240–340 °C range, however above this temperature, the structure collapses and forms MnO_2_ (Figure S2).

### 3.4. Surface Area Analysis Using BET Absorption/Desorption Isotherm

**Figure 3:**
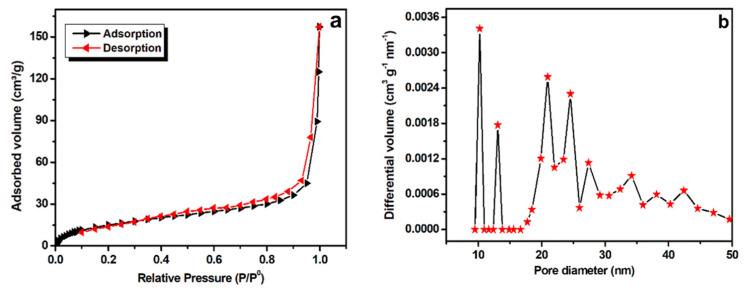
(**a**) BET analysis of **Mn-CP** and (**b**) average pore distribution calculated from BET analysis data.

The surface area of **Mn-CP** was investigated using Brunauer-Emmett-Teller (BET) analysis (Figure 3). From N_2_-sorption isotherms, the specific surface area was calculated to be 60.2 m^2^g^−1^ (Figure 3a), comparable to those of the previously reported analogues [19] and with an average pore size distribution of about 10–50 nm (Figure 3b), suggesting it to be a mesoporous material. The **Mn-CP** high specific surface area, with its mesoporosity, can provide an unobstructed passage of the electrolyte ions to the redox sites, as well as oxygen/hydroxyl diffusion during the catalysis of oxygen reduction and oxygen/hydrogen evolution (see below).

### 3.5. Electrochemical Analysis of Mn-CP

Electrochemical characterization was performed in order to estimate the catalyst (**Mn-CP**) real surface area (RSA). CVs at different polarization rates were recorded in the potential region near the open circuit potential, Figure S3. By plotting Δj = f(ν), where Δj = j_a_ − j_c_ and ν is scan rate in mV s^−1^, the double-layer capacity (C_dl_) was determined to be ca. 0.11 mF cm^−2^. This value is higher than that reported for thermally reduced mesoporous manganese MOF@reduced graphene oxide (rGO) nanocomposites [34], and falls between values previously reported for different Cu complexes (0.29 and 0.065 mF cm^−2^) [35]. The double-layer capacitance is directly proportional to the real surface area (RSE = C_dl_/C_ref_ with C_ref_ being the reference value of capacity per the unit area) and the number of active sites on the electrode surface.

### 3.6. Catalyst Activity of Mn-CP toward ORR, OER and HER in Alkaline Media

**Figure 4:**
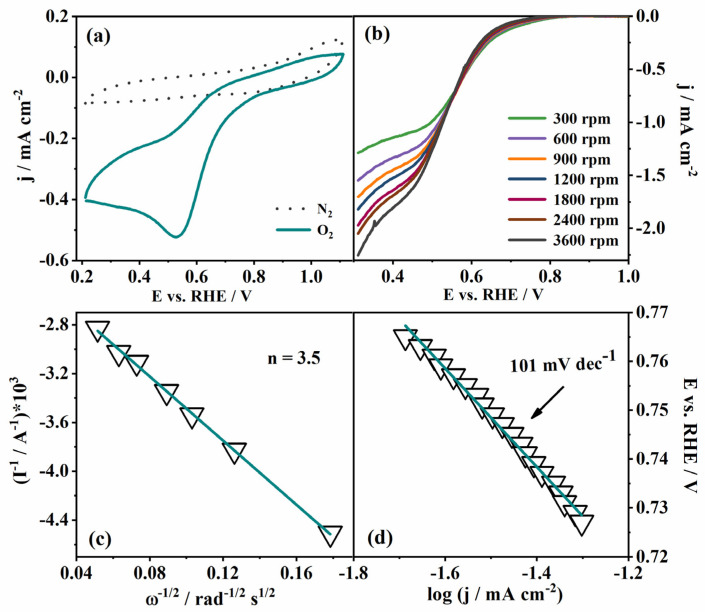
(**a**) CVs of **Mn-CP** in N_2_- and O_2_-saturated 0.1 M KOH, and (**b**) polarization curves at different rotation rates in O_2_-saturated 0.1 M KOH. (**c**) Koutecky–Levich analysis and (**d**) Tafel analysis of the ORR of the **Mn-CP** material.

The electrocatalytic activity towards ORR, OER and HER was first investigated in 0.1 M KOH. CVs in ORR potential region in N_2_- and O_2_-saturated KOH recorded at 0 rpm (Figure 4a) revealed a distinct peak at ca. 0.53 V corresponding to O_2_ reduction. This peak potential value shifted toward a less positive potential by approximately 330 mV when compared to the case of the commercial Pt/C (40 wt.% of Pt) electrocatalyst tested in our previous work [2]. Namely, the oxygen reduction peak appears at ca. 0.86 V in case of commercial Pt/C [2]. Furthermore, Wahab et al. reported that peak potential corresponding to O_2_ reduction of a **Mn-BTC** MOF (BTC = benzene tricarboxyate) on reduced graphene oxide (MnBTC@rGO) shifts from 0.61 V to 0.78 V, with an increase of rGO percentage from 0 to 75%, ref. [36] while Gonen et al. reported a peak potential of 0.72 V for **Mn-BTC** MOF at activated carbon (Mn-BTC@AC) [37]. Thus, a comparison of pristine **Mn-CP** tested herein with those combined with highly conductive, high-surface-area carbon material (rGO, activated carbon) reveals a more positive O_2_ reduction peak potential in cases of carbon material being present. The addition of a rather small amount of Vulcan within this study indeed illustrated its contribution to the increase of **Mn-CP** conductivity, resulting in somewhat higher current densities of **Mn-CP** (with Mn-CP:Vulcan ration 12.5:1) (Figure 4b) compared to pure **Mn-CP**, Figure S4. Still, we want to address the activity of the pristine CP that remains a challenge, whereas there are reports on the use of CPs in the form of composites or as precursors for electrocatalysts [34].

LSVs at different rotation rates were recorded in the same potential region (Figure 4b), and the Koutecky–Levich analysis was performed in order to calculate the number of exchanged electrons, *n*, in the elementary step of oxygen reduction, Figure 4c. The constructed K-L plot is a straight line of good linearity with an *n* value of 3.5 electrons, indicating a mixed oxygen reduction mechanism with both 2e^−^ and 4e^−^ reduction proceeding simultaneously on the catalyst surface. Additionally, the good linearity of the constructed plot suggests a first-order reaction in the electrolyte solution with respect to oxygen concentration [38]. The determined number of electrons exchanged during ORR at **Mn-CP** is similar to that previously reported for ORR at Mn-BTC@AC (*n* = 3.65) [37]. Although the *n* value is somewhat smaller than that of the commercial Pt/C catalyst (*n* = 3.96 was reported in our previous work) [2], this is understandable given that Pt is the best-known electrocatalyst for O_2_-reduction catalysis, but of a notably higher price.

Furthermore, Tafel analysis was performed with 1800 rpm LSV data **(**Figure 4d) in order to determine the Tafel slope, *b*, as one of the key ORR kinetics parameters. The Tafel slope value was found to be 101 mV dec^−1^, which is comparable with other related CP catalysts (Table 1). For example, Wahab and coworkers reported the ORR activity of a Mn-BDC (benzene 1,4-dicarboxylate) compound and its GO (graphene oxide) mixed nanocomposite labelled as MnBDC@rGO [36]. The Tafel slope values of Mn-BDC (138 mV dec^−1^, Table 1) and MnBDC@rGO (93.5 mV dec^−1^, Table 1) are comparable to that of our **Mn-CP** (101 mV dec^−1^). In another case, Chao et al. reported the ORR activity of the Mn^II^ MOF [(Tdc)(4,4′-Bpy)]_n_ (Tdc = thiophene-2,5-dicarboxylate; 4,4′-Bpy = 4,4′-bipyridine), and the Tafel slope value (95 mV dec^−1^, Table 1) [34] also comparable to that of our **Mn-CP**. In addition, our **Mn-CP** displays an even better or comparable ORR activity than some of the Co and Ni-based CPs [39]. The half-wave potential (E_1/2_) was also determined from the LSV study and found to be 0.58 V, which is comparable to the reported ones [34,35,38,40].

**Figure 5:**
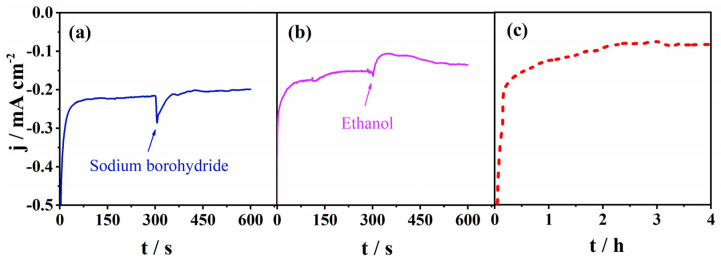
Chronoamperometric responses of **Mn-CP** in O_2_-saturated 0.1 M KOH at a potential of 0.56 V. (**a**) With the addition of 5 mg of sodium borohydride; (**b**) with the addition of 3 M ethanol in the 300th s; (**c**) stability test during 4 h.

Furthermore, in order to determine its potential use in fuel cells, we investigated the selectivity of the **Mn-CP** catalyst towards ORR. The **Mn-CP** catalyst was examined in conditions typical for direct borohydride fuel cells (DBFC) and direct ethanol fuel cells (DEFC). Therefore, sodium borohydride and ethanol were added to 0.1 M KOH saturated with O_2_ to simulate fuel crossover and to study their effect on ORR kinetics. As shown in Figure 5a, the original cathodic current of **Mn-CP** showed a negligible cliff-like drop upon the addition of sodium borohydride to the solution and almost instantaneously regained its value. A somewhat more pronounced drop was observed upon the addition of ethanol, Figure 5b. These results clearly demonstrate that **Mn-CP** possesses a good selectivity toward ORR that is essential for a practical application.

Next, the stability was investigated in O_2_-saturated 0.1 M KOH in the chronoamperometry mode at a constant potential of 0.56 V. The current density during 4 h showed an initial decay and then stabilized, indicating a relatively good stability under ORR polarization conditions, Figure 5c. The initial decay of the current density was higher than expected due to the capacitance current decrease. We believe that this decrease is not solely due to the electrocatalyst’s instability under the specific polarization conditions, but is partially due to the film instability on the conductive support. This could be improved by optimizing the ink composition, which will be presented in a future communication. Moreover, the modular architecture of CPs enables further improvement of the stability of the **Mn-CP** electrocatalyst by varying the chelating ligand (e.g., the donor group), and thus changing the metal–ligand binding energy.

To investigate the multifunctional abilities of the synthesized electrocatalyst, OER and HER studies were also performed in 0.1 M KOH. However, no catalytic activity of the studied catalyst towards HER and OER was observed, as evidenced by low current densities, Figure S5, and high values of the Tafel slope (798 mV dec^−1^ for HER and 545 mV dec^−1^ for OER). An electrochemical impedance spectroscopy (EIS) investigation was performed in order to determine the electrolyte resistance, *R_s_*, and the charge-transfer resistance at the electrocatalyst/electrolyte surface during the electrocatalytic reaction, R_ct_, in both OER and HER potential regions (Figure S6a). *R_s_* values within OER and HER studies were very close (34 Ω in the case of HER and 41 Ω in the case of OER), indicating a minor change in cell geometry and electrode distance during all performed experiments. R_ct_ was found to be as high as ≈7191 Ω for HER and ≈4089 Ω for OER, illuminating a low OER and HER activity of the studied material in 0.1 M KOH.

### 3.7. Catalytic Activity of Mn-CP toward ORR, OER and HER in Acidic Media

The catalytic activity toward ORR in acidic media (0.5 M H_2_SO_4_) was also investigated by performing cyclic voltammetry experiments in N_2_- and O_2_-saturated electrolytes at 0 rpm (Figure S7a), followed by LSV experiments at different rotation speeds (Figure S7b). No measurable difference is observed between current densities in N_2_- and O_2_-saturated 0.5 M H_2_SO_4_, as well as between current densities at 900 rpm and 1200 rpm, indicating that no oxygen reduction occurred in acidic media on the studied catalyst.

**Figure 6:**
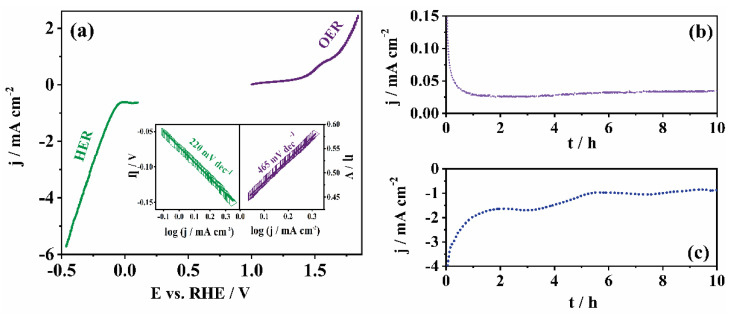
HER and OER polarization curves in 0.5 M H_2_SO_4_ with the corresponding Tafel plots in the inset for **Mn-CP** (**a**). Stability tests under (**b**) OER (constant potential of 1.4 V) and (**c**) HER (constant potential of −0.5 V) conditions in 0.5 M H_2_SO_4_.

LSV experiments performed in OER and HER potential regions in 0.5 M H_2_SO_4_ at 1200 rpm are shown in Figure 6, along with Tafel plots for both reactions in the inset. A higher electrocatalytic activity toward both investigated reactions is observed in acidic media compared to the results in alkaline media (see above), with a notably higher current density recorded.

An earlier onset of both reactions is also observed in acidic media, and is presented as the onset potential in Table 2 together with other key parameters being compared with literature data. It should be noted that literature data regarding OER experiments with Mn-based MOFs in acidic media are very rare, so we opted for comparing it also with similar materials that were tested in alkaline media. Only recently, Singh et al. demonstrated OER catalysis by a pristine Mn MOF (MnTPA) (TPA = terephthalate) [41]. However, they found that MnTPA alone has a very poor OER activity. This is due to the poor electrical conductivity of Mn and the electrically insulating property of the TPA ligand. With the addition of carbon black (CB) and optimizing the MnTPA to CB ratio, they obtained a MnTPA/CB composite (MnTPA:CB ratio as high as 1:2) which achieved a current density of 10 mA cm^−2^ at an overpotential of 539 mV. Additionally, a higher loading of this composite material on the working electrode was used. Thus, although Table 2 might not show a better performance of the herein tested electrocatalyst compared to some reported in the literature, it serves to point out possible directions to take in order to improve the tested electrocatalyst’s performance (making a composite with carbon material certainly being one direction to follow).

Tafel analysis revealed a significantly lower value of Tafel slope for HER in acidic than that in alkaline media (220 mV dec^−1^ vs. 798 mV dec^−1^, respectively), as well as a slightly lower value for OER (465 mV dec^−1^ vs. 545 mV dec^−1^ in acidic and alkaline medium, respectively). However, both values are significantly higher than that of commercial Pt/C (68 mV dec^−1^ for HER and 198 mV dec^−1^ for OER) [43]. This is again expected, because Pt is well known for its ability to catalyze the hydrogen evolution reaction.

Figure S6b presents Nyquist plots in acidic media at both HER and OER potential regions. R_ct_ in OER conditions was observed to be high (ca. 1944 Ω), while under HER conditions the value was significantly lower (ca. 175 Ω). This difference in the charge transfer resistance under OER and HER polarization conditions might account for the higher activity, i.e., higher current densities recorded under HER conditions.

Thus, promising features of the **Mn-CP** electrocatalyst may concern its high specific surface area and high porosity, providing a high number of active sites and a large contact area between those sites and electrolytes, with porosity further enabling easier diffusion of reactants/products. The presence of Mn that can exist in different oxidations states further assists the electrocatalysis as, for instance, sites in higher oxidations states are more active for OER. Still, the low conductivity impedes reaching higher current densities.

Further, stability experiments were performed under both OER and HER conditions in chronoamperometry mode for 10 h. A good stability of the tested catalyst under OER conditions was observed (Figure 6b), as well as a relatively good stability under HER conditions (Figure 6c). This indicates that **Mn-CP** is suitable for applications under harsh conditions in electrolysers. Namely, the current density showed a decrease during the first hour, but then a slight increase was observed during the other 9 h. The HER current densities showed a somewhat more pronounced decrease.

Though Mn-CP showed a lower HER and OER electrocatalytic activity compared to the commercial Pt (40 wt.% Pt) catalyst, i.e., lower current densities (Figure S8a,b, respectively), the promising results and its significantly lower cost make it worthy of further investigation and further studies on the improvement of its catalytic performance.

## 4. Conclusions

The successful design and synthesis of a novel coordination polymer of Mn(II), **Mn-CP**, with an amide-appended ligand are presented. The single-crystal X-ray diffraction analysis revealed that it possesses a 3D structure.

The electrocatalytic activity of **Mn-CP** was tested for ORR, OER and HER in both alkaline and acidic media for the first time. It showed a good performance for ORR in alkaline media, as evidenced by the low Tafel slope and the number of exchanged electrons of 3.5. In addition to the key parameters, the catalyst also showed relatively good stability for 10 h in 0.1 M KOH medium. However, the synthesized material was not active for ORR in an acidic environment.

On the other hand, for HER and OER, the same catalyst showed a better performance (in the form of higher current densities and lower Tafel slopes) in acidic than in alkaline medium; this may be a specially promising finding, as earth-abundant counterparts to noble metal electrocatalys in acidic media are still scarce.

The promising performance of **Mn-CP** may originate from its high specific surface area and high porosity, providing a high number of active sites and easy access of reactants to those sites. The existence of different oxidation states of Mn may enable the materials applications for electrocatalysis of different reactions. At this moment, its low electric conductivity impedes the achievement of higher current densities.

Despite having a lower electrocatalytic activity than a commercial Pt (40 wt.%) catalyst, the lower cost and good redox activity, along with good stability of **Mn-CP**, make this CP an interesting avenue for future investigation and improvement. Further research is already underway in this area.

## Figures and Tables

**Table 1:** Comparison of key parameters of ORR at **Mn-CP** in 0.1 M KOH with literature data ^a^.

Sl. No.	Catalyst	Tafel Slope (mV dec^−1^)	j_d_ (mA cm^−2^)	n	j_k _ (mA cm^−2^)	C_dl _ (mF cm^−2^)	E_1/2 _ (V)	Ref.
1	Mn-CP	101	−1.64	3.5	−0.46	0.11	0.58	This work
2	MnBDC	138	-	-	-	-	0.91	[36]
3	MnBDC@75% rGO	93.5	≈−2.1	-	-	-	0.94	[36]
4	Mn^II^[(Tdc)(4,4′-Bpy)]_n_	95	≈−5.7 at 1600 rpm	3.65	-	-	0.79	[34]
5	Mn-HIB-MOF	-	−4.08	-	-	-	0.74	[40]
6	Ni_3_HITP_2_	237	-	2.46	-	-	0.66	[39]
7	Co_3_HITP_2_	89	5.52	3.97	-	-	0.80	[39]
8	Co_0_._73_Ni_2_._27_HITP_2_	180	-	-	-	-	0.72	[39]
9	Co_01_._27_Ni_1_._73_HITP_2_	148	-	-	-	-	0.75	[39]
10	Co_02_._14_Ni_0_._86_HITP_2_	102	-	-	-	-	0.78	[39]

^a^ j_d_ = diffusion-limited current density, n = number of electrons exchanged during ORR, j_k_ = kinetic current density, C_dl_ = double-layer capacitance, E_1/2_ = half-wave potential. HITP—2,3,6,7,10,11-hexaiminotriphenylene; BDC—benzene-1,4-dicarboxylate; Tdc—thiophene-2,5-dicarboxylate; 4,4′-Bpy-4,4′-bipyridine; HIB—hexaiminobenzene.

**Table 2:** Comparison of HER and OER parameters of the herein studied catalyst with (**Mn-CP**) literature data ^a^.

Catalyst	Reaction	Electrolyte	Tafel Slope (mV dec^−1^)	j_0_ (mA cm^−2^)	j_η_ (mAcm^−2^)	E_onset_ (V)	Ref.
Mn-CP	HER	0.5 M H_2_SO_4_	220	−0.628	−4.808 (0.4 V)	−0.03	This work
Mn-CP	OER	0.5 M H_2_SO_4_	465	0.277	0.948 (0.4 V)	1.59	This work
MnTPA/C	OER	0.05 M H_2_SO_4_	-	-	10 (0.539 V)	-	[41]
MnBDC@75% rGO	OER	1 M KOH	83	-	-	≈1.75	[36]
Mn-HIB-MOF	OER	0.1 M KOH	-	-	10 (0.40 V)	-	[40]
Mn/Fe-HIB-MOF	OER	0.1 M KOH	45		10 (0.28 V)	1.33	[40]
Mn/Co-HIB-MOF	OER	0.1 M KOH			10 (0.40 V)	1.46	[40]
Mn/Ni-HIB-MOF	OER	0.1 M KOH			10 (0.39 V)	1.40	[40]
Mn/Ru-HIB-MOF	OER	0.1 M KOH			10 (0.38 V)	1.43	[40]
TiCP-PCP	HER	0.5 M H_2_SO_4_	142	-	≈−17		[42]
TiCP-PCP	OER	1 M KOH	117	-	-		[42]

^a^ j_0 =_ exchange current density, j_η_ = current density at a given overpotential, E_onset_ = onset potential. TPA = terephthalate; BDC = benzenedicarboxylate; HIB = hexaiminobenzene; PCP = catechol porphyrin.

## Data Availability

Data available on request to the corresponding author.

## Supplementary Materials

The following supporting information can be downloaded at: https://www.mdpi.com/article/10.3390/molecules27217323/s1, Figures S1–S8 containing PXRD, TGA, CVs, polarization curves and Nyquist plots. Crystallographic data and refinement parameters are presented in Table S1 and selected bond distances and angles in Table S2.

## Sample Availability

Samples of the compound [Mn(L)(HCOO)]_n_ is available from the authors.
