# Supplementary material for: Electrocatalytic Behavior of an Amide Functionalized Mn(II) Coordination Polymer on ORR, OER and HER
Source: Molecules. 2022 Oct 28;27(21):7323. doi: 10.3390/molecules27217323 (PMC9655238; doi:10.3390/molecules27217323)
Supplement: Supplementary file 1 [file molecules-27-07323-s001.zip › molecules-1935970-supplementary.pdf]

Electronic Supplementary Information

Electrocatalytic Behavior of an Amide Functionalized Mn(II) Coordination Polymer on ORR, OER and HER

Anup Paul [1,*], Kristina Radinović [2], Susanta Hazra [1], Dušan Mladenović [2], Biljana Šljukić [2,3,*], Rais Ahmad Khan [4], Maria Fátima C. Guedes da Silva [1,5] and Armando J. L. Pombeiro [1,6,*]

[1] Centro de Química Estrutura, Institute of Molecular Sciences, Instituto Superior Técnico, Universidade de Lisboa, Av. Rovisco Pais, 1049-001 Lisboa, Portugal
[2] University of Belgrade, Faculty of Physical Chemistry, Studentski Trg 12-16, 11158 Belgrade, Serbia
[3] Center of Physics and Engineering of Advanced Materials, Instituto Superior Técnico, Universidade de Lisboa, 1049-001 Lisbon, Portugal
[4] Department of Chemistry, College of Science, King Saud University, P.O. Box 2455, Riyadh 11451, Saudi Arabia
[5] Departamento de Engenharia Química, Instituto Superior Técnico, Universidade de Lisboa, Av. Rovisco Pais, 1049-001 Lisboa, Portugal
[6] Research Institute of Chemistry, Peoples' Friendship University of Russia (RUDN University), 6 Miklukho-Maklaya Street, 117198 Moscow, Russia
* Correspondence: anuppaul@tecnico.ulisboa.pt (A.P.); biljana.paunkovic@tecnico.ulisboa.pt (B.Š.); pombeiro@tecnico.ulisboa.pt (A.J.L.P.)

Contents:

Figure S1. Simulated and experimental PXRD of **Mn-CP**.

Figure S2. TGA of **Mn-CP**.

Figure S3. CVs in N_2-saturated 0.1 M KOH at different scan rates with the corresponding double-layer capacitance plots inset for **Mn-CP**.

Figure S4. OER in 0.1 M KOH and HER and OER in in 0.5 M H_2SO_4 polarization curves of pure Vulcan (a,b) and pure **Mn-CP** (c,d) with the corresponding Tafel plots in inset.

Figure S5. HER and OER polarization curves of **Mn-CP** in 0.1 M KOH with the corresponding Tafel plots in inset.

Figure S6. Nyquist plots of **Mn-CP** in (a) 0.1 M KOH, and (b) 0.5 M H_2SO_4.

Figure S7. (a) CVs of **Mn-CP** in N_2- and O_2-saturated 0.5 M H_2SO_4 and (b) polarization curves at 900 and 1200 rpm in O_2-saturated 0.5 M H_2SO_4.

Figure S8. HER (a) and OER (b) polarization curves of commercial Pt/C (40 wt.% Pt) catalyst in 0.5 M H_2SO_4 with the corresponding Tafel plots in inset.

Table S1. Crystallographic data for **Mn-CP**.

Table S2. Selected structural parameters [distances (Å) and angles (º)] in **Mn-CP**.

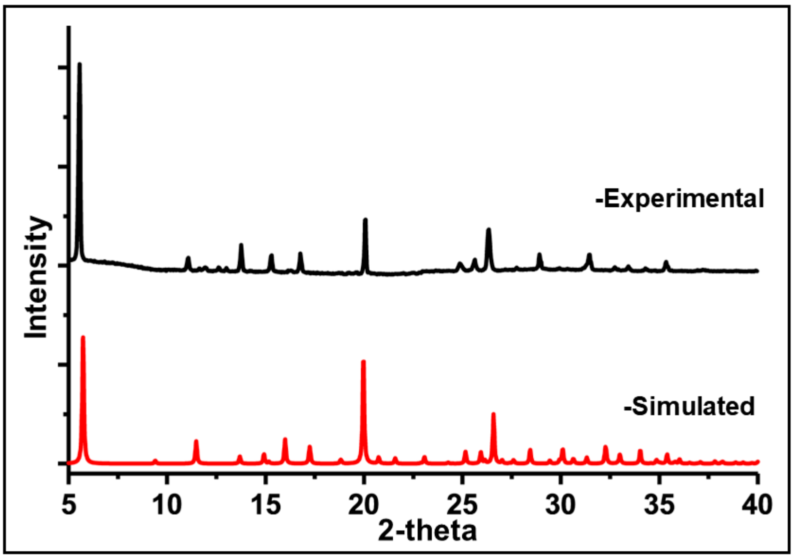

Figure S1. Simulated and experimental PXRD of **Mn-CP.**

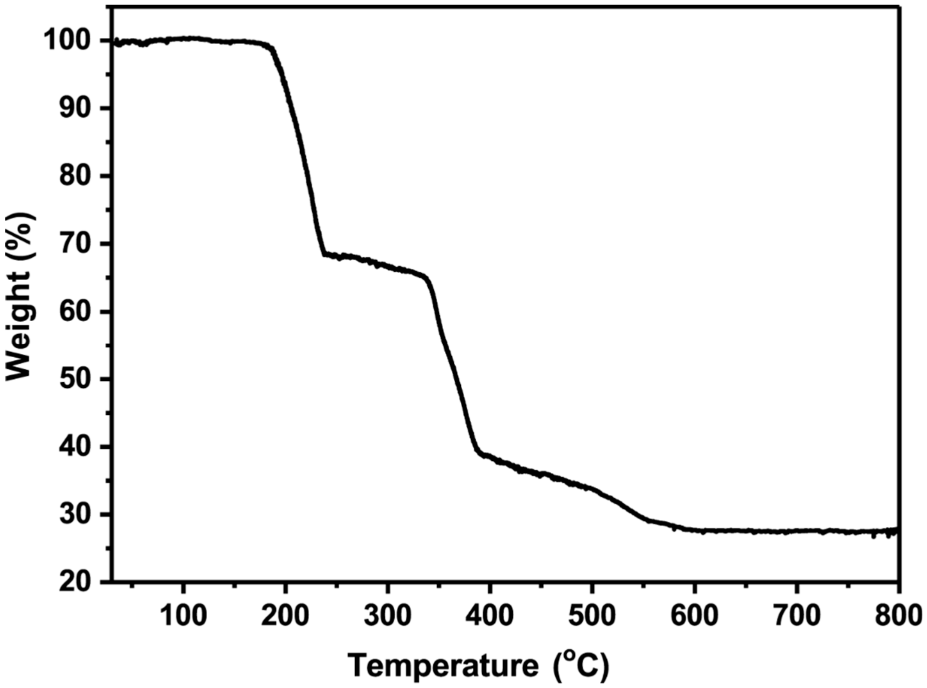

Figure S2. TGA of **Mn-CP**.

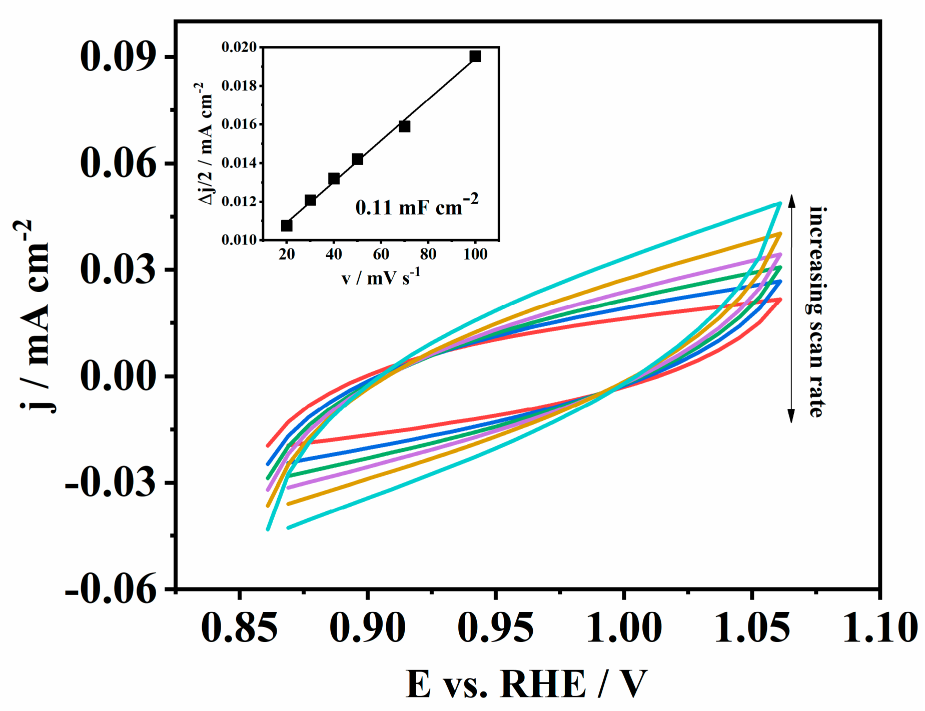

Figure S3. CVs in N_2-saturated 0.1 M KOH at different scan rates with the corresponding double-layer capacitance plots inset for **Mn-CP**.

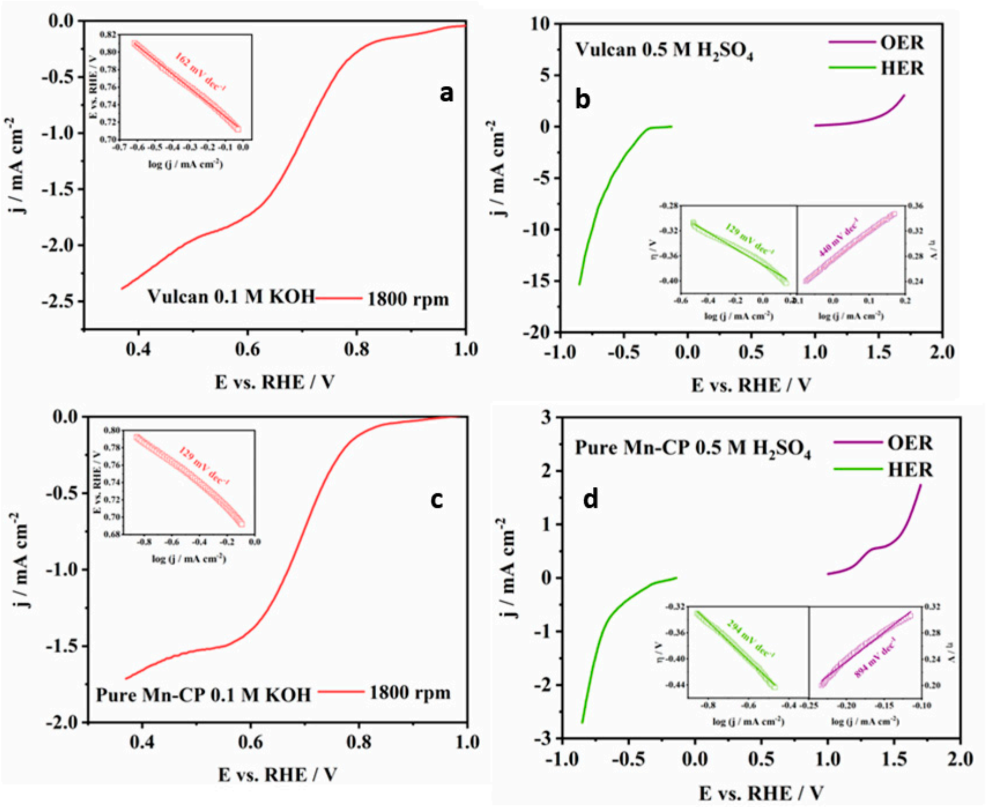

Figure S4. OER in 0.1 M KOH and HER and OER in 0.5 M H_2SO_4 polarization curves of pure Vulcan (a,b) and pure **Mn-CP** (c,d) with the corresponding Tafel plots in inset.

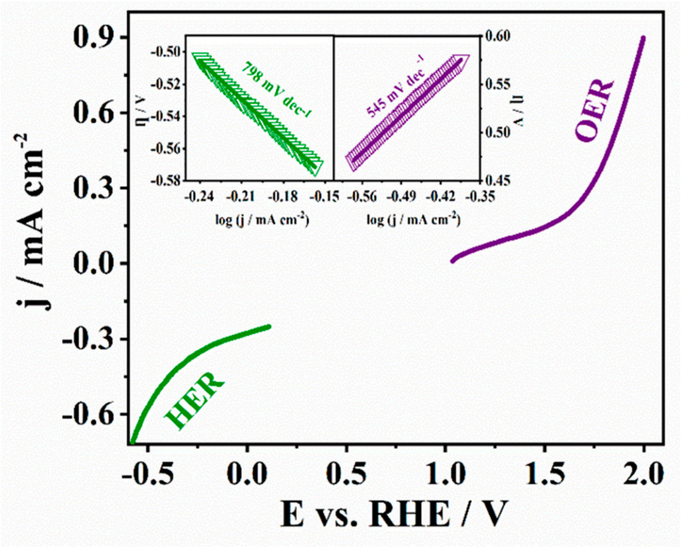

Figure S5. HER and OER polarization curves of **Mn-CP** in 0.1 M KOH with the corresponding Tafel plots in inset.

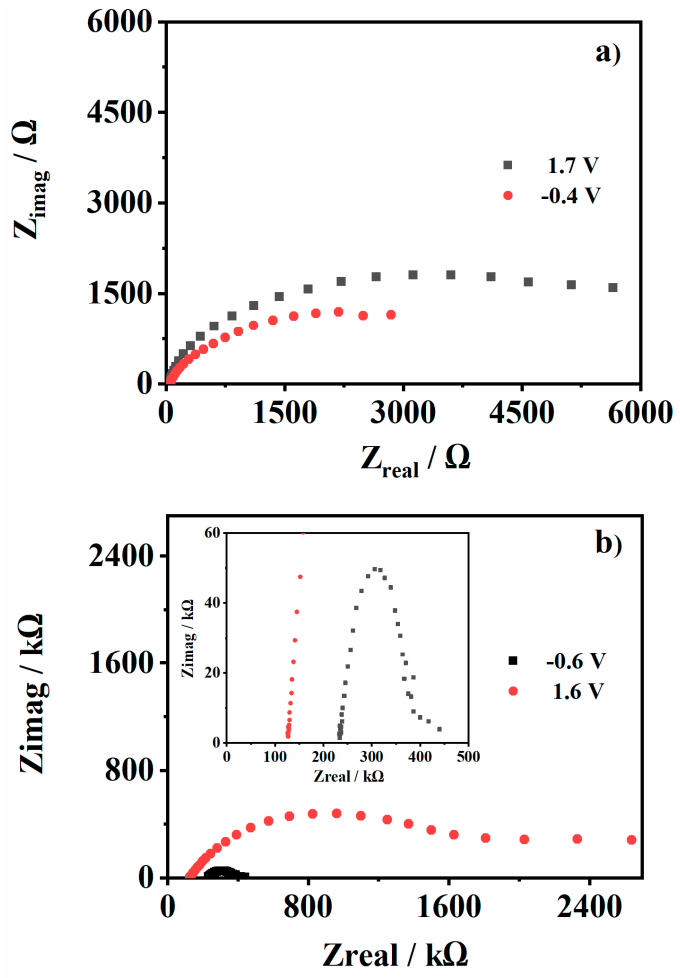

Figure S6. Nyquist plots of **Mn-CP** in (a) 0.1 M KOH, and (b) 0.5 M H_2SO_4.

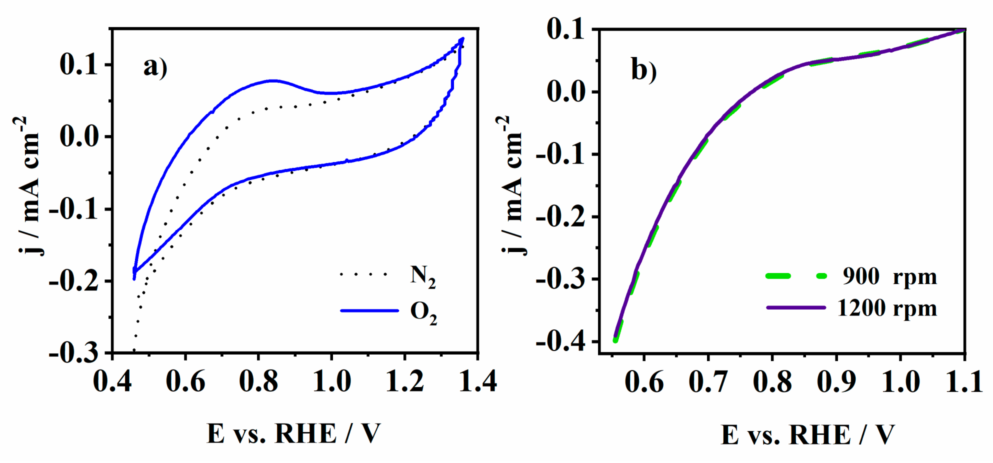

Figure S7. (a) CVs of **Mn-CP** in N_2- and O_2-saturated 0.5 M H_2SO_4 and (b) polarization curves at 900 and 1200 rpm in O_2-saturated 0.5 M H_2SO_4.

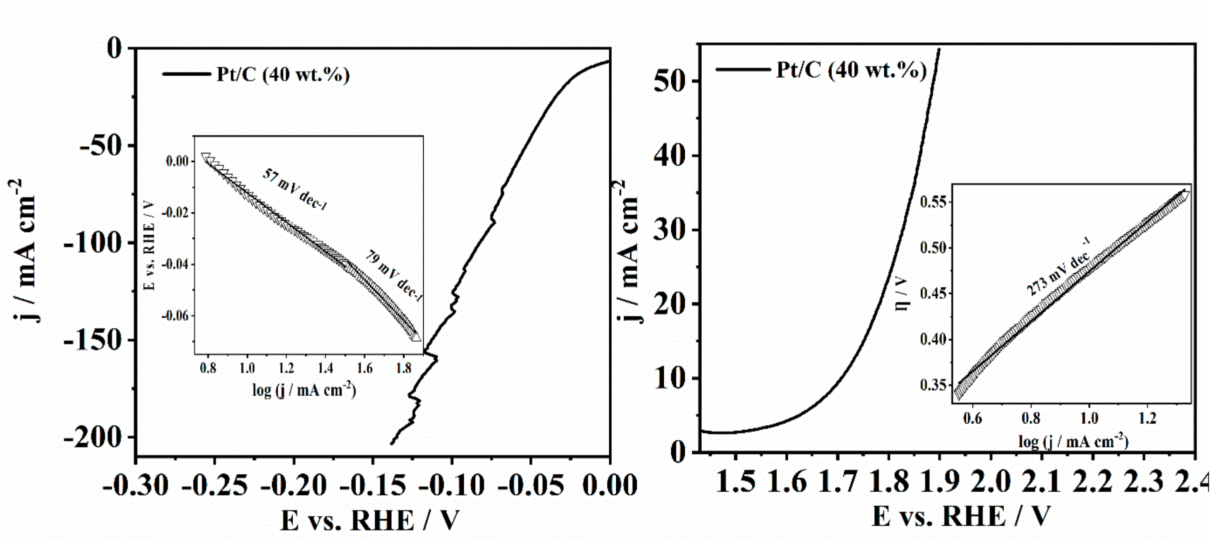

Figure S8. HER (a) and OER (b) polarization curves of commercial Pt/C (40 wt.% Pt) catalyst in 0.5 M H_2SO_4 with the corresponding Tafel plots in inset.

Table S1. Crystallographic data for **Mn-CP**.

	**Mn-CP**
Formula	$C_{14}H_{10}MnN_2O_5$
FW	341.18
Crystal system	Monoclinic
Space group	$P21/c$
a/Å	15.7858(7)
b/Å	11.8665(6)
c/Å	6.8661(3)
β/°	102.5610(10)
V/Å^3	1255.39(10)
Z	4
θ/°	3.15 – 25.40
μ (Mo Kα)/mm^{-1}	1.080
ρ_{calcd}/g cm^{-3}	1.805
F(000)	692
Index ranges	$-19<h<19$
	$-14<k<14$
	$-8<l<8$
Rfs. collected	9896
Rfs. unique/observed	2319/2310
R_{int}	0.0910
R_1/ wR_2 [I > 2σ(I)]	0.0183/0.0439
R_1/ wR_2 [for all F_o^2]	0.0184/0.0442
GOF on F^2	1.001

Table S2. Selected structural parameters [distances (Å) and angles (º)] in **Mn-CP**.

In the L– ligand	
C=O_{amide}	1.2223(18)
C–N_{amide}	1.4045(18)
∠between the l.s. planes of the aromatic rings	37.32
Around the metal centre	
Mn–$N_{pyridyl}$	2.2663(12)
Mn–$O_{carboxylate}$	2.1495(10)<br>2.1510(12)<br>2.1578(13)<br>2.2141(9)<br>2.2571(13)
∠$N_{pyridyl}$–Mn–$O_{caboxylate}$ (*trans*)	168.88(4)
∠$O_{formate}$–Mn–$O_{formate}$ (*trans*)	171.65(4)
∠$O_{formate}$–Mn–$O_{caboxylate}$ (*trans*)	164.16(4)
